# *Bubalus bubalis*: A Short Story

**DOI:** 10.3389/fvets.2020.570413

**Published:** 2020-12-01

**Authors:** Antonio Humberto Hamad Minervino, Marco Zava, Domenico Vecchio, Antonio Borghese

**Affiliations:** ^1^Laboratory of Animal Health, LARSANA, Federal University of Western Pará, UFOPA, Santarém, Brazil; ^2^Argentine Buffalo Breeders Association, Buenos Aires, Argentina; ^3^Italian National Reference Centre on Water Buffalo Farming and Productions Hygiene and Technologies (CReNBuf), Istituto Zooprofilattico Sperimentale del Mezzogiorno, Portici, Italy; ^4^International Buffalo Federation – IBF, FAO/ESCORENA Buffalo Network, Animal Production Research Institute, Rome, Italy

**Keywords:** water buffalo, bubaline, breeds, dairy buffalo, buffalo population

## Abstract

The domestic buffalo (*Bubalus bubalis*), also known as water buffalo or Asian buffalo to prevent confusion with the American bison (*Bison bison*), wrongly named buffalo in North America, comprises two subspecies: the river buffalo (*B. bubalis bubalis*) and the swamp buffalo (*B. bubalis kerebau*). The swamp buffalo has a consistent phenotype and is considered as one type, even if many breeds are recognized within it; conversely, the river buffalo subspecies has many breeds. We found limited information available regarding the worldwide distribution of buffaloes. The best estimate is that 208,098,759 buffalo head are distributed in 77 countries in five continents. In this review, we presented the basic aspects of the water buffalo and unraveled the buffalo path followed from the origin of the species to its current global distribution. We reviewed several data sources to provide a better estimate of the world buffalo count and distribution.

## The *Bubalus* Genus

**Figure 1:**
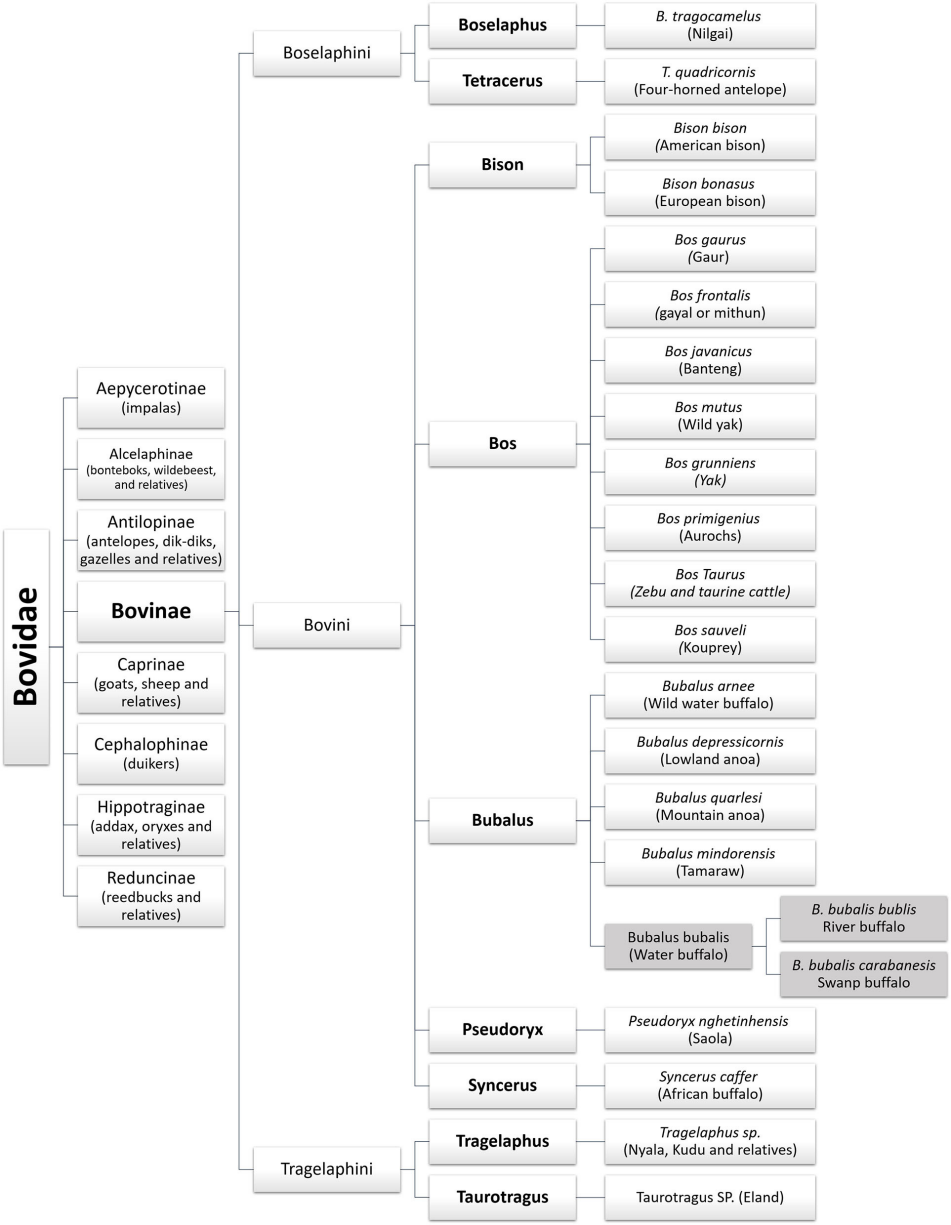
Zoological classification of the water buffalo in the Bovinae family.

Bovids are the largest family within the Artiodactyla order. The name bovines or Bovinae Gray, 1821 refers to a subfamily of the Bovidae family, which includes nine genera: *Bos* (cattle), *Poephagus* (yak), *Bison* (bison), *Syncerus* (African buffalo), *Boselaphus* (nilgai), *Pseudoryx* (saola), *Tetracerus* (four-horned antelope), *Tragelaphus* (kudu and relatives), and *Bubalus* (domestic buffalo) (1–3). Figure 1 depicts the zoological classification of the Bovinae subfamily.

**Figure 2:**
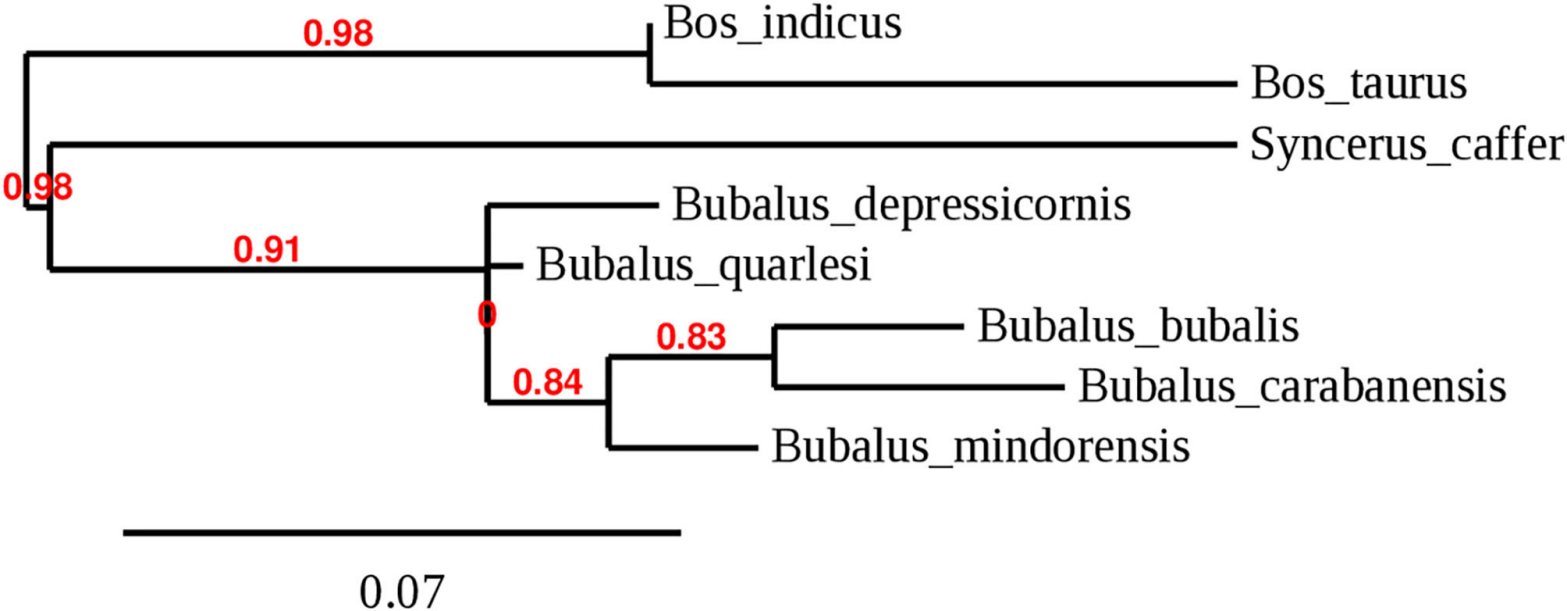
Phylogenetic tree of the genus *Bubalus* based on D-loop region complementing with IOC and cytochrome B.

*Bubalus*, a genus of the Bovidae family, was distributed widely in Europe and Asia during the Pleistocene. Wild animals of this genus include the wild Asian buffalo (*Bubalus arnee*) that originates in northern India and lives in marshes and in the jungle. This is a very large animal, reaching a height of up to 200 cm and a weight of up to 1,000 kg; it can be either gray-black, dark gray, or dark brown and has large horns that are separated at the base (4). This species was distributed across Asia but is now considered endangered (5). The anoa, which is endemic to Indonesia, comprises two species: the lowland anoa (*B. depressicornis*) and the mountain anoa (*B. quarlesi*); however, there is some debate on whether they are the same or distinct species (6). The anoa inhabits only Indonesia, had 46 chromosomes, and is a small animal (100 cm tall) with thin and straight horns (25 cm long). It is conserved in zoos and never used for draft work or for food production and lives in mountains or in lowland forests (7). The anoa was divided in *B. depressicornis* and *B. quarlesi* based on the environment it inhabited. *Bubalus mindorensis* is found only on the island of Mindoro in the Philippines; therefore, it is also called tamaraw or Mindoro buffalo. It is a small animal (100 cm tall) with short and strong horns. It is a critically endangered species from the Philippines with only 220–300 mature animals remaining (8). Figure 2 shows the phylogenetic tree based on the D-loop region of several species of the Bovini tribe, including the *Bubalus* genus.

## The Water Buffalo

The water buffalo or Asian buffalo (*Bubalus bubalis*) is a domesticated species that can be confused by the general population with the wild American bison (*Bison bison*), incorrectly called Buffalo in North America, and occasionally, with the African Buffalo (*Syncerus caffer*), which are two unrelated species of wild animals. The water buffalo comprises two subspecies: the river buffalo (*B. bubalis bubalis*) and the swamp buffalo (*B. bubalis kerebau*), which are genetically distinct with different chromosome numbers (50 and 48, respectively), and distinct morphology, physiology, and productive and reproductive performances (9). The two subspecies are interfertile, with their progeny containing 49 chromosomes. Male crossbred progeny may sometimes present fertility problems, while female progeny exhibits longer calving intervals (10); however, this only applies in the case of further backcrossing.

The morphology of the two subspecies differs considerably. River buffaloes are usually larger than swamp buffaloes and weigh between 450 and 1,000 kg. Most breeds have curled horns and are mainly found in India, Pakistan, and some European, western Asian, and American countries. The river buffalo is primarily reared for dairy production, especially in Asia and Europe, but is also used for meat production or as a dual-purpose animal as well as for draft purposes (4, 11). Swamp buffaloes are smaller and lighter than river buffaloes and normally weigh between 325 and 450 kg. Swamp buffaloes are reared mainly for draft purposes; however, they can also yield high amounts of milk (up to 600 kg of milk per year) (11). Swamp buffaloes are predominant in Southeast Asia and Australia (12). A few animals can also be found in the north-eastern states of India (13).

Buffaloes can be affected by the same diseases and parasites as cattle; the extent to which they are affected varies drastically depending on the country, region, and production system. Buffalo breeds have varying degrees of tick resistance; however, buffaloes are infested by the louse *Haematopinus tuberculatus* that is specific to them (14). Brucellosis, tuberculosis, leptospirosis, bovine viral diarrhea, fasciolosis, foot-and-mouth disease, and protozoal infections have important economic impacts on the water buffalo industry. Regarding public health, water buffaloes are involved in the transmission of schistosomiasis to humans (15, 16). A recent report showed that buffaloes may play some part in Q fever epidemiology, as *Coxiella burnetii* was detected in buffalo milk (17).

The buffalo has a higher capacity to convert feed with poor nutritional value, and a high capacity for adaptation and survival in different environments with distinct topography, climate, and vegetation (18). The water buffalo is used as a draft animal in Asia, owing to its strength; however, it is also used as a riding animal on Marajó Island, Brazil. Water buffaloes are highly adapted to hot and wet floodplains and play an important role in the economy of many tropical and subtropical countries, as they are used for meat and milk production and as draft animals (19). Water buffaloes have relatively lower heat tolerance than other livestock species owing to their dark coat color and their inadequately dispersed sweat glands, which result in a less efficient evaporative cooling system (20). Recent molecular studies have shown that buffalo breeds historically raised in a hot climate may have developed higher heat tolerance (21). Buffaloes have higher longevity than cattle (they can surpass 30 years of age, while maintaining their reproductive capacity until they are 18–25 years old). This docile, intelligent, and curious animal (Supplementary Video 2) is farmed in several countries worldwide, mainly for its good quality meat (which has better nutritional composition compared to that of cattle) and its high-fat milk (11, 19, 22, 23).

## Water Buffalo Domestication

The two distinct water buffalo subspecies (river and swamp) descended from different populations of the wild Asian water buffalo that diverged some 900,000 years before present (BP) and evolved in separate geographic regions. The wild Asian buffalo is considered the most probable ancestor of the water buffalo, and the most accepted hypothesis supports two independent domestication events from river and swamp buffalos. Recent molecular studies support that domestication started from a wild ancestor, *Swamp-like*, that was distributed across mainland Asia and has long since diverged into the existing population of *B. arnee* (9, 24). Compared with *Bos taurus* (10,000 BP), the buffalo domestication occurred recently, ~3,000–7,000 years BP.

The domestication of river buffaloes probably occurred at ~6,300 yr BP in north-western India, from where domesticated river buffaloes migrated west, across south-western Asia, Egypt, and Anatolia, and reached the Balkans and the Italian peninsula (25). Phylogenetic analyses and archaeological evidence suggest that the river buffalo was domesticated in an atypical and complex manner, including multiple maternal lineages, with the successive introgression of wild animals to domestic stocks (26). River buffalo populations show a weaker phylogeographic structure but higher phenotypic diversity, thereby resulting in more breeds (27). After domestication, the water buffalo was introduced in Egypt and Italy by the Arab invasion around the eighth century. Buffaloes were also introduced in the Balkans and Turkey by crusaders and by the Selgiukid invasion during the expansion of the Ottoman Empire in the fifteenth century (7).

The swamp buffalo was most likely domesticated near the border between China and Indochina at ~3,000–7,000 years BP, spreading across Southeast Asia. Swamp buffaloes have strong phenotypic uniformity, but molecular studies show a higher diversity of maternal and paternal lineages, strong genetic differentiation affected by geography, and a lack of gene flow (27, 28). One large study with 46 buffalo populations in seven Asian countries revealed an abundant genetic diversity and clear phylogeographic structure in swamp buffalo populations (29). One exception is considered a swamp buffalo lineage from China that has a complex pattern of diversity, thereby suggesting the presence of a long-term, strong gene flow, probably due to extensive migrations of buffaloes alongside human movements (30). A recent study with buffalo whole genome showed similar genetic patterns in both cattle and buffalo breeds, an example of convergent domestication down to the same mutation but which occurred independently (31). Phylogenetic analysis revealed that Chinese swamp buffaloes could be divided into two distinct lineages, A and B. Of the two lineages, lineage A was predominant across all populations. For predominant lineage A, Southwestern buffalo populations possess the highest genetic diversity among the three hypothesized domestication centers (Southeastern, Central, and Southwestern China), suggesting that Southwestern China is the most likely location for the domestication of lineage A. However, a complex pattern of diversity is detected for lineage B, preventing the unambiguous pinpointing of the exact location of the domestication center and suggesting the presence of a long-term, strong gene flow among swamp buffalo populations caused by extensive migrations of buffaloes and frequent human movements along the Yangtze River throughout history (31).

## Water Buffalo Breeds

**Figure 3:**
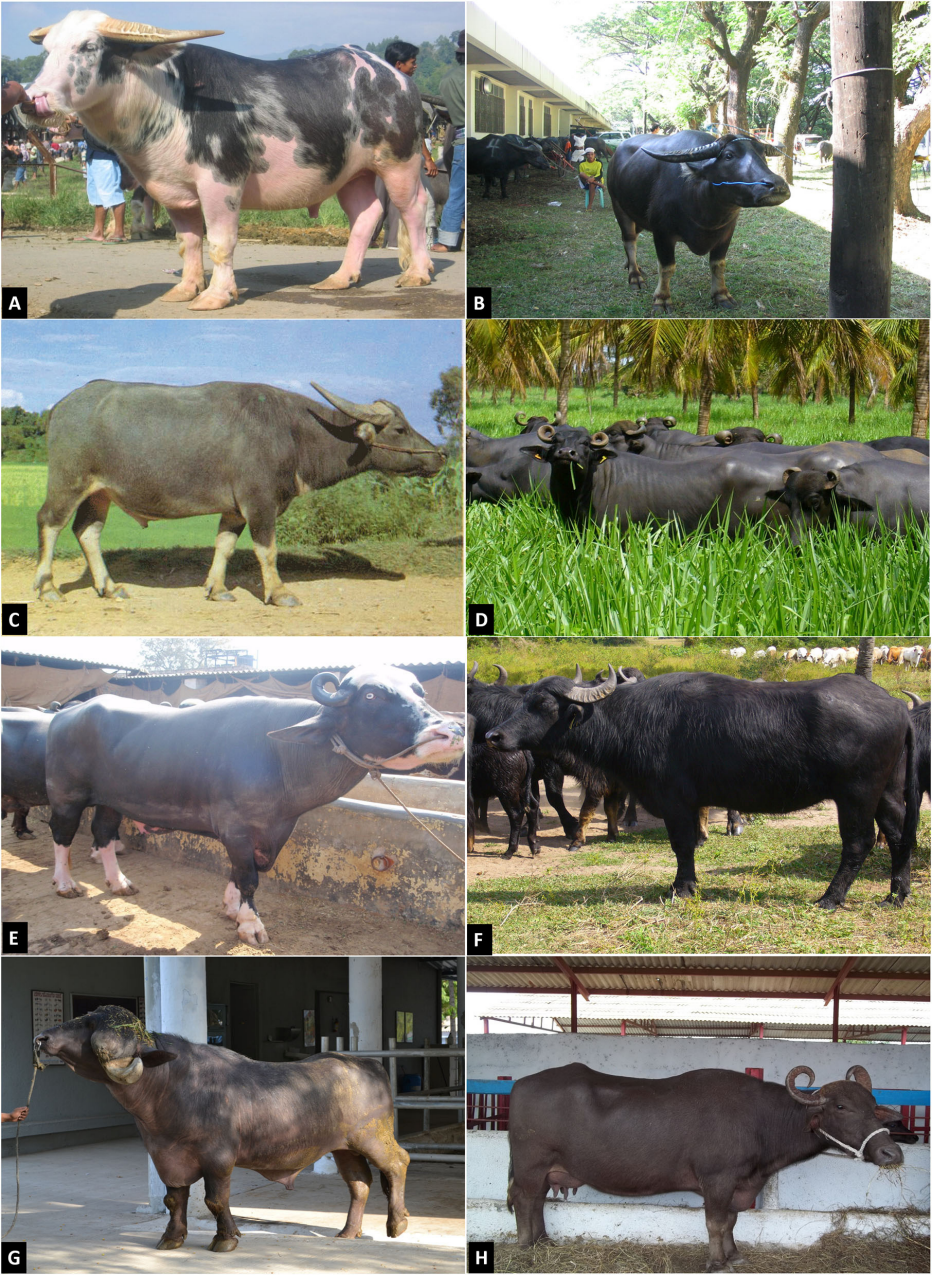
Buffalo breeds worldwide. **(A)** Sulawesi breed spotted animal (Indonesia); **(B)** the Carabao breed from Philippines; **(C)** Fuling breed in China; **(D)** Murrah buffaloes herd from São Paulo, Brazil; **(E)** Nili-Ravi breed at Pakistan; **(F)** Mediterranean Italy breed raised in Mozambique; **(G)** Jaffarabadi bull in Brazil; **(H)** Buffalypso breed from Trinidad and Tobago. Source: Castro, S.R., Borghese (7).

As mentioned above, the water buffalo comprises two subspecies, the swamp and the river buffalo. The swamp buffalo has a consistent phenotype and is considered as one type; however, separation in breeds still occurs, especially depending on the geographic location. Conversely, the river buffalo comprises several breeds. According to a Food and Agriculture Organization (FAO) report based on data provided by countries, there are 123 buffalo breeds, 90 in Asia alone, many of which are local breeds, with only 15 breeds reported as transboundary (32). There is a lot of variation in horn conformation. In many cases, it is one of the indicators of the degree of purity in different breeds. Figure 3 shows photos of swamp and river buffaloes, while Table 1 presents a list of the most important breeds and recognized populations of water buffalo.

**Table 1:** Breeds or recognized population of water buffalo worldwide.

**Breed or recognized population**	**Subspecies**	**Local**	**Purpose**
Anatolian	River	Turkey	Milk and meat
Azeri or Caucasian	River	Azerbaijan, Georgia Armenia, Iran	Milk and meat
Azikheli	River	Khwazakhela, Swat, Pakistan	Milk
Bangladeshi breed	River	West Bangladesh	Milk and draft
Bangladeshi Surti	River	Central Bangladesh	Milk
Bhadawari	River	Uttar Pradesh, India	Milk and draft
Binhu	Swamp	Hunan, China	Draft
Buffalypso	River	Trinidad and Tobago, Cuba	Selected for meat and draft
Carpathian	River	Transylvania, Romania	Milk and meat
Dongliu	Swamp	Anhui, China	Draft
Egyptian	River	Egypt	Milk and meat
Enshi	Swamp	Hubei, China	Draft
Fuling	Swamp	Sichuan, China	Draft
Fuzhong	Swamp	Guanxi, China	Draft
Jafarabadi	River	Gujarat, India and Brazil	Milk and meat
Jerangi	River	Andhra Pradesh, India	Draft
Kalahandi	River	Orissa, India	Draft and milk
Kerbau Papangan	Swamp	South Sumatra marshlands	Milk and meat
Khuzestani	River	Iraq and Iran	Milk and meat
Kundi	River	Sindh, South Pakistan	Milk
Lime	River	Nepal mountains and river valleys	Triple aptitude
Manda	River	Andhra Pradesh, India	Milk and draft
Mazandarani	River	Iran	Milk and meat
Mediterranean	River	East Europe, Macedonia, Greece, Serbia, Albania	Triple aptitude
Mediterranean Italian	River	Italy and export (live animals and semen)	Selected for mozzarella and processing industry
Meshana	River	Gujarat, India	Milk
Mesopotamian	River	Iraqi marshlands	Milk and meat
Murrah	River	India and export (live animals and semen)	Selected for milk
Murrah, Bulgarian	River	Bulgaria	Milk
Nagpuri	River	Madhya Pradesh, India	Milk and meat
Nili-Ravi	River	Pakistan and Punjab, India	Selected for milk
Original Far East Buffalo	Swamp	Thailand, Vietnam, Laos, Cambodia, Myanmar, Malaysia	Triple aptitude, family animal, rice fields
Pandharpuri	River	Maharashtra, India	Milk
Parkote	River	Nepal mountains and river valleys	Triple aptitude
Philippines Carabao	Swamp	Philippines rice fields	Triple aptitude
Primitive Bangladeshi	Swamp	East Bangladesh	Draft and meat
Sambalpuri	River	Madhya Pradesh, India	Milk
Shanghai	Swamp	Shanghai, China	Draft
South Kanara	River	Mangalore west coast	Draft
Sulawesi spotted	Swamp	TanaToraja Sulawesi, Indonesia	Sacrificed during ceremonies
Surti	River	Gujarat and Rajasthan, India	Milk
Tarai	River	Uttar and Madhya Pradesh, India	Milk and meat
Toda	River	Madras, India	Milk and for ceremonies

The swamp buffalo, despite having a consistent phenotype, had somatic and physiological differences that can be noted depending on the climate and the land, owing to the total isolation of herds. The swamp buffaloes of Sulawesi that live in the jungle are completely different from the Sumatran swamp breed that lives in lagoons. The Sulawesi breed is clearly a meat animal, strong and muscular with a big head, and very appreciated in ceremonies, particularly if it is a spotted animal (Figure 3A), a characteristic that adds value to the animal. Sumatran buffaloes are lighter animals, with thin heads, and are mostly used for milk purposes (7).

The Carabao breed that is native to the Philippines is a swamp-type buffalo (Figure 3B) used as a draft animal in small-hold, land-based agriculture. Carabao buffaloes have low, wide, and heavy built bodies. Their body coloration varies from light gray to slate gray. Their horns are sickle-shaped or curved backward toward the neck (11). On the island of Marajó, Pará, Brazil, these animals are used for meat production. They are brown-gray, have white spots on their legs, are developed for work, and are suitable for meat production, while their milk production capacity is usually poor (33).

The Chinese buffalo is a swamp-type buffalo, used mostly for draft work, and, depending on the region, comprises a diverse array of breeds, which amount to 18 local breeds. The most important of these breeds are the Fuling breed (Figure 3C) living in the Sichuan mountains, and the Binhu breed, living in Hunan province; these are the breeds with the highest populations, which amount to more than 400,000 head each. Other breeds include the Fuzhong and Xilin breeds from Guanxi province, Enshi breed from Hubei, Dongliu breed from Anhui province, and the Shanghai breed. Swamp breeds in China are mainly used for draft purposes as they are very efficient in the marshlands, particularly in rice fields, and have limited milk production capacity (10, 28).

Swamp subspecies are also dominant in Southeast Asian countries such as Malaysia, Laos, Cambodia, Vietnam, Myanmar, and Thailand. In these countries, the buffalo is an important part of the crop production system, as it is the main draft power source for land preparation, supplies fresh organic manure for cultivation, and is highly adapted to living in different habitats. Australia imported swamp animals from Asia as the management systems in the country are extensive (11, 34).

The river subspecies breeds have been named according to Mason (35). There are three international river breeds that are widely distributed worldwide, owing to their high genetic potential for milk production: Murrah, Nili-Ravi, and the Italian Mediterranean (36).

Murrah is the most represented and well-known buffalo breed in the world. It is selected for milk production in northwest India and is known for its curled horns (Figure 3D). Its name is a Hindu word that means spiral and derives from the shape of its horns. Its skin and hair color is jet black. White spots are not accepted, except at the end of the tail. These are massive and stocky animals with heavy bones. Their horns are short and tightly curled. This breed originated in the center of Haryana and spread across India. It has been exported to several countries worldwide, such as Brazil, Bulgaria, and East Asian countries (10, 37). In Bulgaria, the only buffalo breed is the Bulgarian Murrah, which resulted from crossbreeding the Indian Murrah and the local Mediterranean breed. Many animals have been exported from Bulgaria to neighboring countries, such as Romania and Germany, and to distant countries, such as Brazil and Venezuela. Murrah buffaloes were exported to China and Indonesia to increase milk availability, as the local breeds are of the swamp type with low milk production capacities; the same applies for many Asian countries, in which the original buffalo population is of the swamp type (19, 37).

The second most represented breed in the world is the Nili-Ravi (Figure 3E). This is the most important livestock breed in Pakistan with a head count higher than 10,000,000 in Punjab, while it is also present in India (38). This breed resembles the Murrah breed but has white markings on its extremities and walled eyes; its horns are less curled than those of the Murrah breed. The skin color of the breed is generally black, but there are some albino animals, brown, spotted, or with clear eyes (38, 39). Nili-Ravi is the most important dairy buffalo breed in Asia and is selected for this purpose, with its milk yield reaching 3,050 kg (40). The Kundi breed is widespread in the Sindh region in south Pakistan (10). The Azikheli buffalo is an undocumented buffalo breed from Khwazakhela, Swat, Pakistan. It has a small body size, brown-colored coat, and horns lying flat, bending laterally, and directed backward and slightly upward without twisting (41).

The Mediterranean buffalo descended from the river buffalo introduced to Europe from India during the Arab occupation of the eighth century in Sicily and in the south of Italy. The Italian breed is now classified as Italian Mediterranean breed (Figure 3F) as it was selected for 60 years for milk purposes and is clearly a dairy breed. This breed was later exported to several countries in America and Asia, with a large export of Mediterranean semen from Italy. Buffaloes were also introduced in east Europe by crusaders in the twelfth century and later with the Turkish invasion of the Ottoman Empire (fifteenth century). These breeds are different from the Italian Mediterranean breed. Balkan breeds such as the Carpathian or the Macedonian are draft animals, used in carriages, and they are smaller in size and with low milk yield capacity (36).

European Mediterranean animals have black, brown, and dark gray coats, while Italian Mediterranean animals are exclusively black. Their horns are flat at the bottom and face backward, and their points face upward and inward. The Mediterranean is a compact breed with a deep and wide chest, but short back and rump. The udder is medium-sized with squarely placed quarters and halves and with cylindrical teats (10, 36). The breed's size, weight, and productivity vary greatly depending on the genetics, environment, and management system. The average daily milk yield varies widely and depends on factors such as genetics and the feeding system. It can range from 3 to 4 kg milk/day for poorly fed animals up to 15 kg milk/day in intensive production systems (10). The Italian Mediterranean breed has high milk yield and quality, as it was selected for the creation of a dairy breed specialized for the cheese industry, with a genealogical book established in 1980, and the National Association of Buffalo Breeders (ANASB) established in 1979. The use of the milk data recorded for females registered on the genealogical book, the application of several cycles of progeny testing, and the spread of artificial insemination with the use of semen of proven high genetic value created an improved dairy breed, with many females producing more than 5,000 kg milk/270 days of lactation, to a maximum of 5,600 kg with 8.3 fat and 4.6 protein, as the selection goal was not the total milk yield but mozzarella production (7).

The Jafarabadi breed (Figure 3G) is originally from Gujarat, India. It is a large animal with a massive and long-barreled conformation and black coat. Its horns are long, heavy, begin at the base of the head, face downward, and then curve. The Jararabadi breed is of particular importance in India, with animals being exported to Brazil. In Brazil, the Jafarabadi breed has two varieties: the Gir, which is of medium size and the most widespread variety, and the Palitana, a large breed used in crossing.

The milk yield of the Jafarabadi is good; however, this is also a large animal with high muscularity. Therefore, this breed was selected by many countries in the Americas as a meat-purpose breed. It is possible to find pure Jafarabadi animals or their descendants in Brazil, Colombia, and other South American countries, where this breed showed great adaptability to marshlands and other environments, and a high affinity for meat production. Additionally, the Jafarabadi was the basis for the creation of the Buffalypso breed in Trinidad and Tobago and in Cuba (7).

The Buffalypso is the typical buffalo breed of Trinidad and Tobago (Figure 3H) derived from the Jafarabadi breed imported in Trinidad in 1905. In 1948, Steve Bennet crossed Bhadwari bulls with Jafarabadi cows to create the Buffalypso breed (Buffalo × Calypso, a form of popular Caribbean music), with the purpose of obtaining a meat breed, which is also useful as a draft animal. Subsequently, the Buffalypso was crossed with the Murrah, Surti, Nili, and Nagpuri breeds. It is now also used for milk production (10, 33). The highest Buffalypso head count is in Cuba and Trinidad and Tobago; however, the breed can also be found in several countries such as Venezuela, Costa Rica, Guatemala, the Republic of Honduras, Nicaragua, Brazil, Panama, Mexico, Colombia, USA, and even Taiwan. Historically, Buffalypso is the only breed selected exclusively for meat, as it is a very muscular animal with developed back and rump, brown-colored, and sold in the American market as beef.

Other river breeds in India include the Surti breed in north Gujarat, one of the most important breeds in the Rajasthan and Gujarat regions, with a population higher than 500,000 buffalo head. These animals have two white chevrons on their chest, and their horns are flat and sickle-shaped, directed downward and backward, and then turn upward at the tip to form a hook (13, 42). The Tarai breed amounts to ~1,000,000 buffalo head. These animals have long and flat horns with coils bending backward and upward. This breed is raised in Uttar Pradesh and Madhya Pradesh (4, 10). Other breeds of ~300,000–400,000 buffalo head are the Mehsana (its characteristics are intermediate between the Surti and Murrah breeds), reared between the Mahi and Sabarmati rivers in Gujarat, and Nagpuri, a local breed improved by selection from Indian breeds that have long (50–65 cm) and flat-curved horns (4, 10).

Other breeds with smaller populations are the Bhadawari raised in Uttar Pradesh and Madhya Pradesh, the Jerangi (a small animal) and Manda (each amounting to 100,000 buffalo head), which are breeds raised along the border of Orissa with Andhra Pradesh, Sambalpuri from Bilaspur in Madhya Pradesh, and Toda, which is a large breed raised in the Nilgiri hills of Madras (7). Additionally, there are Pandharpuri, Kalahandi, and South Kanara River buffalo breeds in India.

In Nepal, the Lime breed is believed to have descended from the ancestral wild buffalo, possibly with river-type introgression, and became domesticated in the country (43). The Lime buffalo is found in the mountains, high hills, and hill river valleys in Nepal with a herd population of ~700,000 head, which accounts for ~35% of the total indigenous buffalo population. This is a small-sized breed, with small sickle-shaped horns that are curved toward the neck, and light brown in color with chevrons of gray/white hair below the jaw and around the brisket (7).

The Parkote buffalo is a typical animal of the mid-hills and river valleys in Nepal. However, owing to the traditional crossbreeding with the Lime and, more recently, the Murrah breeds, their pure breed population estimated at 500,000 head is now declining. The Parkote have dark-colored coats, black skin, black muzzles, and no markings on their legs. These animals are medium-sized, with sword-shaped horns directed laterally or toward the back (10, 35).

The native buffaloes in the western part of Bangladesh are of the river type, known as the Bangladeshi breed, and account for ~45% of the total population. The coat color of these animals is normally black, while they have curly horns. Few individuals have spots in their tail switch, and some are brown-colored or have hanging horns. Their average milk yield is ~620 kg in 270 days of lactation. Male buffaloes are used for draft and females for dairy purposes (44).

The Anatolian buffalo that descended from the migratory Indian buffalo (seventh century) has been raised in Turkey for centuries. These animals are black-colored and have long hair, with variations in their tail lengths and frequent white switches. The Anatolian breed has an estimated population of 122,000 and is raised predominately in the Black Sea region, north of middle Anatolia, Thrace, Hatay, Mus, Kars, Diyarbakir, Afyon, and Sivas (45).

The Azeri or Caucasian breed originated in the Indo valley and descended from the Indian buffalo. Archeological evidence suggests that buffaloes were raised in Iran (Lorestan region), around the ninth century B.C., as buffalo heads have been found engraved on a bronze stick from the same period (11, 46). This is a black-colored breed, with short horns growing backward and a population size of ~600,000 head. It is found in Iran, Azerbaijan, and along the Caspian Sea; it was widespread in Georgia and Armenia until 1940, but then its numbers declined (47).

The Khuzestan buffalo (sometimes called Khuzestani) is a breed amounting to hundreds of thousands head raised in Iraq and Iran. They are large animals, probably the biggest buffalo breed in the Near East, with short horns pointing upward and forming a ring at the end (7, 46). Raised mostly for milk purposes, the average milk production of these animals is ~1,950 kg of milk within 210–240 days of lactation, with a reported average of 8.6 kg/head/day for Khuzestan buffaloes (48). The Azeri and Khuzestan buffaloes, the most common indigenous breeds of Iran, present genomic regions associated with adaptation to different environments resulting from divergent selection in different regions (21, 49). In Iran, there is also the Mazandarani breed raised mainly in the Mazandran province (in the north region of the county) with 4,000 individuals (47).

The Egyptian breed was introduced to Egypt from India, Iran, and Iraq, probably around the seventh century B.C. The differences between the Egyptian buffalo types depend only on the environment. This breed is very important in the dairy industry in Egypt, as it produces 45% of the milk consumed in the country (50). These animals are blackish gray in color, with long and narrow heads, and horns varying in format (from lyre to sword-shaped) (7).

## Water Buffalo Herd

The most robust information regarding the buffalo population is available by the FAO and is collected exclusively from official governmental institution records (51). When there is an absence of official records, the FAO statistics do not count the buffalo herds in that country, which produces a lack of consolidated information regarding the number of countries with buffaloes and the number of buffalo herds worldwide. For instance, FAO statistics indicate that only three countries in the Americas have buffalo populations (Brazil, Trinidad and Tobago, and Suriname), excluding two large buffalo herds in America, Colombia, and Venezuela. Therefore, we conducted an extensive literature review to provide a more accurate count of buffalo herds and to establish the buffalo distribution in the different continents.

The worldwide water buffalo population reported by the FAO in 2017 was ~201 million head with an expansion to 206.6 million in 2018, showing an increase of 2.8% in 1 year. The FAO data includes 43 countries with buffalo populations but did not present an actual herd number in two of those listed countries, Australia and Singapore. There are some reports of buffaloes distributed in 129 countries, first mentioned by Iamartino et al. (52) and replicated by Rahmaninia et al. (47), but this information is inaccurate. The number 129 represented the total number of countries involved in the FAO report and does not necessarily mean that buffaloes exist in each of those countries. Thus, taking as a starting point these FAO data from 2018 and researching recent literature, we retrieved a list of 77 countries that have buffalo herds, with an estimation of 208,098,759 head. Table 2 presents our aggregate data from buffalo herds worldwide. Supplementary Table 1 provides detailed information regarding the source of the buffalo head count presented.

**Table 2:** Current data on buffalo population worldwide.

**Region/country**	**Water buffalo population^†^**
**Worldwide**	**208,098,759**
**Africa (%)**	**3,509,256 (1.69%)**
Egypt	3,506,061
Mauritius	25
Mozambique	1,000
South Africa	170
Tanzania	2,000
**Americas (%)**	**2,562,711 (1.23%)**
Argentina	121,276
Belize	632
Bolivia	35,000
Brazil	1,390,066
Canada	1,120
Chile	100
Colombia	336,417
Costa Rica	20,000
Cuba	60,000
Ecuador	10,000
El Salvador	250
Guatemala	5,000
Honduras	1,500
Mexico	45,000
Nicaragua	800
Panama	4,000
Paraguay	15,000
Peru	1,500
Suriname	905
Trinidad and Tobago	6,145
United States of America	7,000
Uruguay	1,000
Venezuela	500,000
**Asia (%)**	**201,428,230 (96.79%)**
Armenia	717
Azerbaijan	176,195
Bangladesh	1,485,000
Bhutan	531
Brunei	2,319
Cambodia	651,945
China	27,116,250
East Timor	125,760
Georgia	18,361
Hong Kong	329
India	114,151,770
Indonesia	894,278
Iran	199,000
Iraq	300,000
Jordan	95
Kazakhstan	10,414
Laos	1,200,040
Malaysia	117,707
Myanmar	3,790,031
Nepal	5,277,819
Pakistan	38,848,000
Philippines	2,882,655
Sri Lanka	308,790
Syria	8,000
Taiwan	2,057
Tajikistan	15,351
Thailand	1,258,272
Turkey	161,439
Vietnam	2,425,105
**Europe (%)**	**460,795 (0.22%)**
Albania	95
Bulgaria	12,809
Germany	9,613
Greece	9,239
Hungary	1,000
Italy	402,796
Kosovo	400
North Macedonia	643
Poland	69
Romania	14,000
Russia	5,311
Serbia	1,000
Switzerland	1,200
United Kingdom	2,500
Ukraine	120
**Oceania (%)**	**137,767 (0.07%)**
Australia	133,000
Guam	94
Micronesia	173
New Zealand	1,000
Papua New Guinea	3,500

† *The majority of data was obtained at FAOSTAT (51). Additional sources: (7, 27, 33, 47, 53–77). Detailed methodology is presented in the Supplementary Table 1*.

Notably, the absence of official data regarding buffalo herds in most countries mandates the retrieval of estimates from buffalo studies. Even developed countries (high-income economies), such as Canada or New Zealand, do not have any data available regarding their buffalo populations, according to their respective ministries of agriculture. Despite the absence of official information, it was possible to retrieve data on the global distribution and head count of buffaloes with some degree of confidence, mainly owing to our contacts with breeders' associations and with fellow scientists around the globe. Our data may be unreliable for two major countries, Australia and Venezuela. Australia, in particular, has two distinct herds, one feral water buffalo herd and one domesticated herd, which have been increasing in numbers, owing to the use of animals for dairy purposes and the increase in animal imports in recent years (78). Recent reports estimate the population in the Northern Territory of Australia to be ~70,000 animals; however, the herd sizes of Western Australia and Queensland are not known (79). The number of feral buffaloes in Australia was recently estimated at 120,000 animals by Lemcke (2019) *apud* Zhang (27). The Australian Buffalo Industry Council estimates that there are ~15,000–20,000 domestic water buffaloes in the country; however, a recent report projected 13,000 farmed buffaloes (27). Therefore, the best estimate is for 133,000 buffalo head in Australia (27).

**Figure 4:**
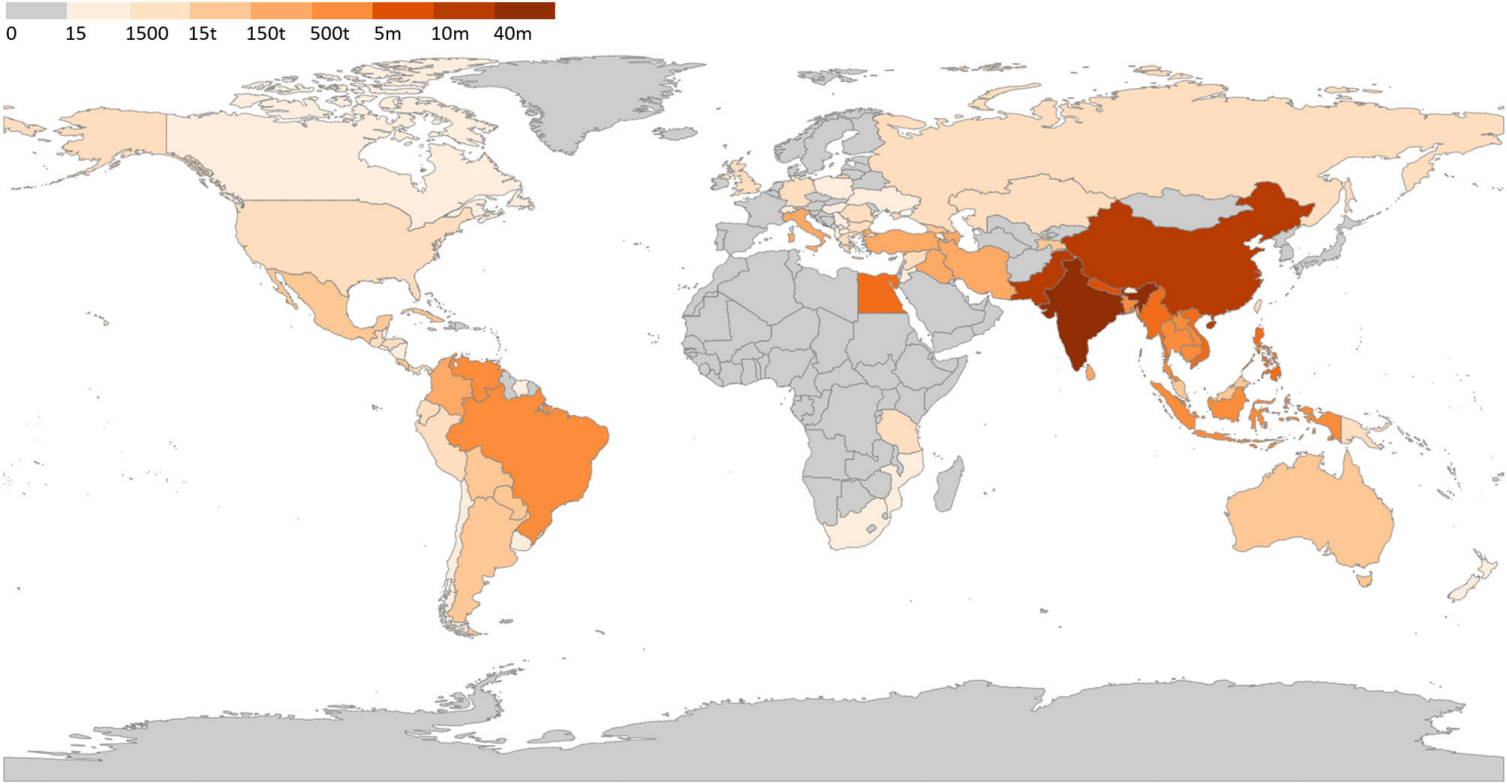
Water buffalo population worldwide. Legend indicate number of head in each country; t, thousand; m, million. Data source available at Supplementary Table 1.

Venezuela has an increasing buffalo herd that is mostly raised in the wetland of the Llanos region, representing the second largest herd in the Americas, after Brazil (80). The herd evolved from 180,000 (81) to 350,000 animals (33). A recent report of the Venezuelan Buffalo Breeders Association reported the existence of 500,000 animals belonging to more than 190 farmers, with unverified reports suggesting that there are more than 1.8 million buffalo head in Venezuela (53). Considering the absence of official data, we considered the data reported in the aforementioned recent report. Considering the current economic situation in Venezuela, it is uncertain if this population still stands. Based on our aggregate data, we created a world map with the estimated distribution of water buffalo herds (Figure 4).

**Figure 5:**
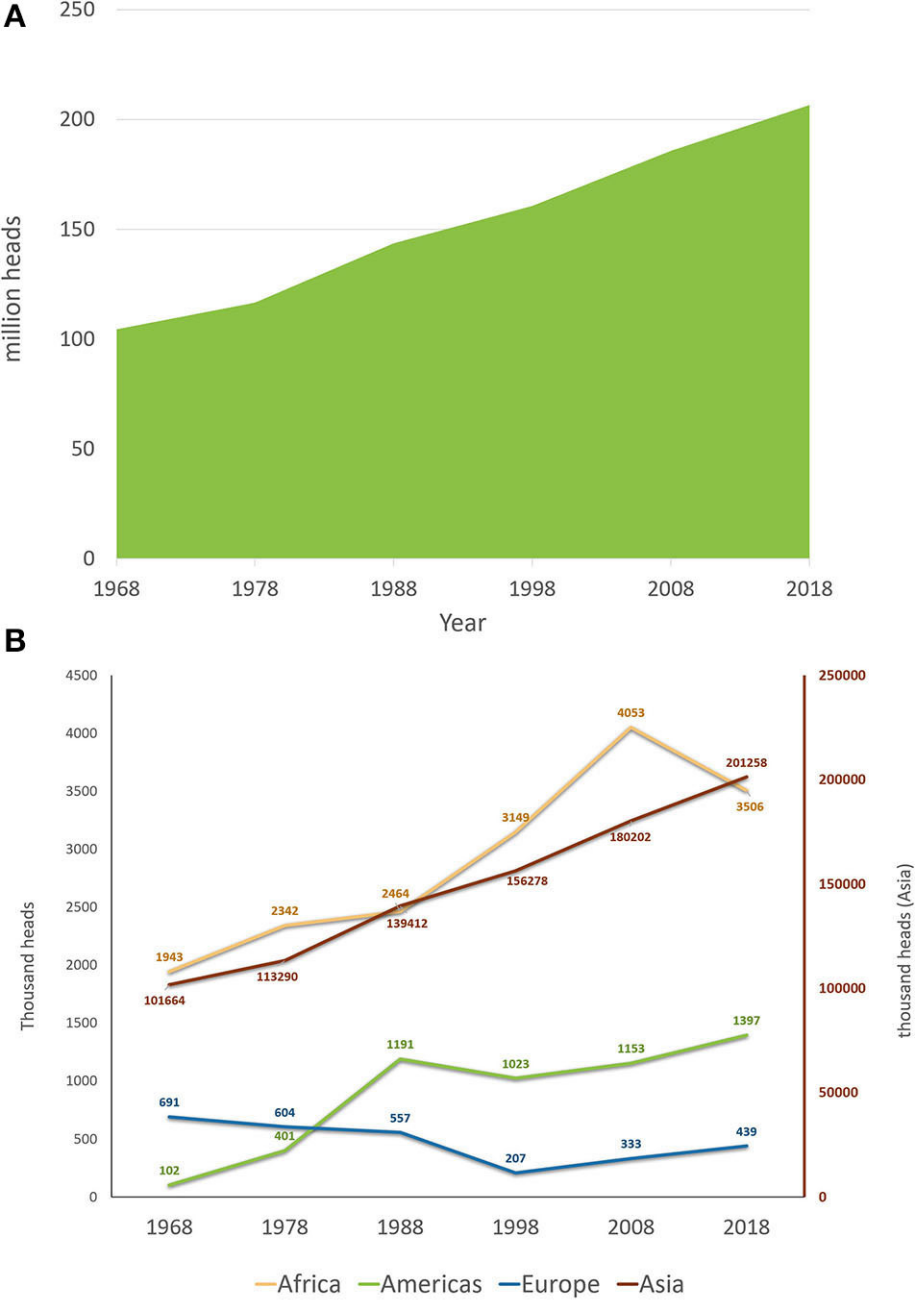
Worldwide buffalo population trends: **(A)** worldwide buffalo population from the past 50 years (1968–2018); **(B)** worldwide buffalo population from the past 50 years (1968–2018) separated by continent. The right red axis indicates the count for Asia. America, Africa, and Europe are represented in the left axis. Source FAOSTAT (51).

Buffalo herds have been subjected to major changes in recent history. Supplementary Video 1 presents the evolution of buffalo herds in different countries from 1962 to 2017 using FAO data (51). Regarding the buffalo population trends, Figure 5A presents the water buffalo herd evolution in the past 50 years using data from 1968 to 2018. During these 50 years, buffalo herds worldwide almost doubled, with a population increase of 97.9%. Compared with cattle, buffalo herds had a superior trend of increase (considering the percentages), as cattle herds increased by ~40% from 1968 to 2018. Although the buffalo herd had a greater proportional increase, the cattle population is far more numerous. The cattle herd in 1968 was around 1.06 billion reaching 1.49 billion in 2018, with an increase in the past 50 years of 425 million head (51). An evaluation of each 10-year period from 1968 to 2018 showed that the global buffalo count increased steadily at a mean rate of 14.7 ± 5.0% per 10 years. The 10-year increase was ~11% for the periods that ended in 1978, 1998, and 2018, with sharper increases in 1988 (23%) and 2008 (15.6%). An evaluation of the buffalo population trends from the past 50 years, depending on the continent (Africa, the Americas, Asia, and Europe) is shown in Figure 5B. It can be noted that the buffalo population of Asia has a constantly increasing trend, while the buffalo population of Europe decreased until 1998, at which point it started to increase, but without reaching the original herd count of 1968. This phenomenon could be attributed to the fact that in Europe, only Italy constantly increased its buffalo population because of its dairy breed, cheese industry, and a market with a strong economy; on the other hand, in Eastern European countries, where buffaloes were used as draft animals for land work or carriages and had low milk yield, they were replaced by Friesian cows and mechanization. Similarly, in Asia, India, and Pakistan, the dairy breed populations increased rapidly, while in southeastern countries, the swamp buffalo populations decreased. Countries in the Americas and Africa experienced increasing trends; however, both areas experienced a 10-year period when their buffalo populations decreased (1988–1998 in the Americas and 2008–2018 in Africa).

## Buffalo Milk Production

In many countries, buffalo breeding has a family character in which the herds are represented by a few animals used for subsistence. In many societies, buffalo milk is acquiring more and more visibility linked to its intrinsic value as a dairy product, as it can not only be consumed directly, as is the case in the Asian continent, but can also be transformed into dairy products of high commercial demand (36, 40). According to FAO data, the buffalo milk production accounted for ~15.14% of the global fresh milk production in 2018, while cattle milk accounted for more than 80% (Table 3). Supplementary Video 3 presents the buffalo milk production (in tons of milk per year) in different countries from 1962 to 2017 using FAO data (51).

**Table 3:** Buffalo, camel, cow, goat, and sheep milk whole fresh produced in 2018 for each region.

**Milk, whole fresh^†^**		**Buffalo**	**Camel**	**Cow**	**Goat**	**Sheep**	**Total**
World	Tons	127,658,734	3,137,071	683,217,056	18,712,088	10,631,058	843,356,007
	%	15.14%	0.37%	81.01%	2.22%	1.26%	100.00%
Asia	Tons	124,958,493	262,233	213,201,098	10,627,509	4,924,398	353,973,731
	%	35.30%	0.07%	60.23%	3.00%	1.39%	100.00%
America	Tons	189,739	NA	184,304,156	779,806	90,871	185,364,572
	%	0.10%	NA	99.43%	0.42%	0.05%	100.00%
Europe	Tons	390,137	76	220,377,066	2,722,332	3,168,166	226,657,777
	%	0.17%	0.00%	97.23%	1.20%	1.40%	100.00%
Oceania	Tons	NA	NA	30,706,258	39	NA	30,706,297
	%	–	–	100.00%	0.00%	–	100.00%
Africa	Tons	2,120,365	2,874,762	34,628,478	4,582,402	2,447,623	46,653,630
		4.54%	6.16%	74.22%	9.82%	5.25%	100.00%

† *Data from 2018 (51). The percentage (%) of animal species milk was calculated according to each geographical area. NA, not available*.

The comparison of these percentages within each continent produces interesting scenarios. For example, in Asia where buffalo milk accounts for 35.30% of the total milk production, buffaloes are the major milk-producing animal in India and Pakistan; additionally, there are higher populations of dairy buffaloes than dairy cows in Egypt and Nepal (51). The top 10 largest producers of buffalo milk worldwide (India, Pakistan, China, Egypt, Nepal, Italy, Myanmar, Iran, Colombia, and Brazil) are responsible for 97.59% of the global production (Table 4). It should be considered that, given the lack of production data for over 50 of the 77 countries in which buffaloes are bred, these values represent an estimate of the real production potential of the species; in fact, of 208 million buffaloes, 7.48 million are bred in countries in which there are no reported data for buffalo milk production.

**Table 4:** Current data on buffalo milk production worldwide.

**Region/country**	**Milk production^α^**	**%^β^**	**References**
**Worldwide**	**127,658,734**	**100.00%**	
**Africa**	**2,120,365**	**1.66%**	†
Egypt	2,120,365	100.00%	†
**Americas**	**189,739**	**0.15%**	
Brazil	87,472	46.10%	Data estimated (82)
Colombia	102,267	53.90%	Data estimated (76)
**Asia**	**124,958,493**	**97.88%**	
Bangladesh	35,691	0.03%	†
Bhutan	282	0.00%	†
Brunei	184	0.00%	†
China	3,003,323	2.40%	†
Georgia	6,186	0.00%	†
India	91,817,140	73.48%	Official data^γ^
Indonesia	71,166	0.06%	†
Iran	129,904	0.10%	†
Iraq	49,893	0.04%	†
Malaysia	8,190	0.01%	†
Myanmar	193,841	0.16%	†
Nepal	1,338,277	1.07%	Official data^γ^
Pakistan	28,109,000	22.49%	Official data^γ^
Sri Lanka	85,914	0.07%	Official data^γ^
Syrian	6,300	0.01%	Official data^γ^
Turkey	75,742	0.06%	Official data^γ^
Viet Nam	27,460	0.02%	†
**Europe**	**390,137**	**0.31%**	
Albania	12	0.00%	†
Bulgaria	11,753	3.01%	Official data^γ^
Greece	402	0.10%	†
Italy	377,970	96.88%	Official data (65)

α *Water buffalo milk production worldwide in tons (2018 data)*.

β *Percentage is related to the contribution of each continent to the global buffalo milk production and from each country to their continent total milk production*.

† *FAO data based on imputation methodology (51)*.

γ *Official data retrieved from Faostat 2019 (51)*.

To date, only Italy has established an official system for the traceability of milk production and the entire buffalo supply chain since 2014 (83), which provides real-time information on milk production. The establishment of this system was favored by the economic value that the supply chain represents for the country. In fact, Italy, which ranks fifth globally in terms of its milk production, produces 97% of the buffalo milk produced in Europe (Table 4); only the buffalo mozzarella that is protected under the EU's Protected Designation of Origin (PDO) scheme, is estimated at a sales value of 766 million euros (84).

According to FAO data, the global buffalo milk production increased by 32.57% from 2011 to 2018 (from 96 to 127 million tons); on the contrary, cattle milk increased by 10.67% (from 617 to 683 million tons). Many factors that characterize the various breeding activities worldwide make it very complex to estimate the actual production of fresh buffalo milk. The breed, production purpose (milk, meat, work), breeding type (extensive and intensive), feeding techniques, seasonality at different latitudes, age at first birth, weaning techniques, reproductive techniques applied, lactation duration, milking of the subjects, genetic selection applied, and the longevity owing to economic sustainability linked to the commercial value of the milk are some of these factors (42, 85).

There are many factors that affect the milk yield in different species; breed, genetic background, season and period of calving, health status, and environmental factors such as feeding, climate conditions, and welfare are some of these factors. These factors are also important in buffalo species; however, the genetic potential is the most important factor, as exemplified in the Italian Mediterranean, Murrah, and Nili-Ravi breeds. A lot of work has underwent in selecting the most appropriate female lines by recording animals and creating genetic centers with selected bulls for the spread of artificial insemination. Milk production during lactation is a continuous physiological function. In buffalo species, the lactation curve, which is a graphical representation of milk yield during lactation, shows a rapid increase, peaks, reaches a plateau phase, and declines gradually until the end of lactation (86). In economic terms, the most important phase is the persistence of lactation, which means the ability to maintain milk production at high levels after the peak of lactation (87). Genetic and environmental influences on the persistence of lactation were studied in Indian Murrah buffaloes by Geetha et al. (88) and in Nili-Ravi buffaloes by Chaudhry et al. (89), who showed the effect of parity and of lactation length on persistency. The effects of parity, age, and calving season were also studied in the Bulgarian (90) and the Italian Mediterranean breeds (91, 92).

Compared to cow's milk, buffalo milk is approximately twice as high in fat content and ~30% higher in total solids; therefore, if the calculation was made on a dry matter basis, the importance of buffalo milk worldwide would be even greater (93). In terms of energy, 1 kg of buffalo milk equals 5.10 Mj, which is much higher than the 2.90 Mj/kg of 1 kg of cow's milk (94). The water buffalo is of the most productive domestic animals, has a longer productive life when compared to cattle, and is economically important, especially for small-scale producers in developing countries. Given its characteristics of resilience and adaptability to tropical climates (95), the buffalo represents an important food source of animal origin.

Buffalo milk production in several countries is driven for processing and for the cheese industry. The mozzarella cheese is recognized and largely consumed worldwide as an adulteration of the original *Mozzarella* cheese, which is made exclusively from buffalo milk. Only in 1993, was mozzarella from buffalo recognized as “*Mozzarella di Bufala Campana* PDO.” The PDO certification pertains to buffaloes bred in the Campania and Lazio regions of Italy that produce the milk used for the production of the famous mozzarella cheese; what this essentially means is that this cheese has to be produced in the defined areas only from fresh buffalo milk from the Italian Mediterranean breed, registered in the Buffalo Genealogical Book (36). The mozzarella industry in Italy resulted from 34,990 recorded females of the Italian Mediterranean breed, which account for ~30% of the total dairy buffalo population (this percentage does not exist in any other country) and have a mean production of 2,356 kg milk in 270 days of lactation, with 8% fat and 4.63% protein (96).

## Conclusion

There is a surprising lack of data regarding buffalo herds worldwide. In this study, we reviewed the literature and established a more appropriate buffalo distribution and population count. Our results showed that buffaloes are present in 77 countries in five continents with a population of more than 208 million head. Considering buffalo characteristics such as rusticity and productivity, we believe that buffalo production should be expanded worldwide, especially in developing countries with adequate natural conditions. This amazing and subutilized animal can become more productive, and should be promoted as a target species to be used in smallholder production systems. Governments worldwide must look carefully to this amazing animal and start developing projects to introduce this species in regions with favorable conditions, in order to substitute cattle herds.

## Author Contributions

All the authors conceived, research, write, and revised this manuscript.

## Conflict of Interest

The authors declare that the research was conducted in the absence of any commercial or financial relationships that could be construed as a potential conflict of interest.

## Supplementary Material

Supplementary Table 1 Sources and methodology for the aggregate data of the world population of water buffalo (*Bubalus bubalis*) per country.

Supplementary Video 1 This video presents the buffalo herd evolution (in number of animals) in the different countries from 1962 to 2017 using the data from FAO (51). Video produced through FlourishTM platform (Kiln Enterprises Ltd, London, UK). Available online at: https://flourish.studio/.

Supplementary Video 2 Herd of docile and curious mixed-breed buffaloes in Santarém, Amazon, Brazil.

Supplementary Video 3 This video presents the buffalo milk production (in tons of milk per year) in the different countries from 1962 to 2017 using the data from FAO (51). Video produced through FlourishTM platform (Kiln Enterprises Ltd, London, UK). Available online at: https://flourish.studio/.
