# Supplementary material for: Bubalus bubalis: A Short Story
Source: Front Vet Sci. 2020 Dec 1;7:570413. doi: 10.3389/fvets.2020.570413 (PMC7736047; doi:10.3389/fvets.2020.570413)
Supplement: Supplementary Table 1 — Sources and methodology for the aggregate data of the world population of water buffalo (Bubalus bubalis) per country. [file Table_1.DOCX]

Table S1. Buffalo population herd count with respective sources.

| **Name** | **Region** | **Buffalo population** | **Source*** |
| --- | --- | --- | --- |
| Egypt | Africa | 3506061 | FAO data based on imputation methodology(1) |
| Mauritius | Africa | 25 | FAO data based on imputation methodology |
| Mozambique | Africa | 1000 | (2) |
| South Africa | Africa | 170 | (3) |
| Tanzania | Africa | 2000 | (2) |
| Argentina | Americas | 121276 | (4) |
| Belize | Americas | 632 | (5) |
| Bolivia | Americas | 35000 | (5) Updated by author, 2020 |
| Brazil | Americas | 1390066 | Official data |
| Canada | Americas | 1120 | (5) Updated by author, 2020 |
| Chile | Americas | 100 | (5) Updated by author, 2020 |
| Colombia | Americas | 336417 | (6) |
| Costa Rica | Americas | 20000 | (7) |
| Cuba | Americas | 60000 | (8) |
| Ecuador | Americas | 10000 | (9) |
| El Salvador | Americas | 250 | (10) |
| Guatemala | Americas | 5000 | (11) |
| Honduras | Americas | 1500 | (12) |
| Mexico | Americas | 45000 | (13) |
| Nicaragua | Americas | 800 | (5) Updated by author, 2020 |
| Panama | Americas | 4000 | (5) Updated by author, 2020 |
| Paraguay | Americas | 15000 | (14) |
| Peru | Americas | 1500 | (5) Updated by author, 2020 |
| Suriname | Americas | 905 | Official data |
| Trinidad and Tobago | Americas | 6145 | FAO data based on imputation methodology |
| United States of America | Americas | 7000 | (15) |
| Uruguay | Americas | 1000 | (5) Updated by author, 2020 |
| Venezuela | Americas | 500000 | (16) |
| Armenia | Asia | 717 | Official data |
| Azerbaijan | Asia | 176195 | Official data |
| Bangladesh | Asia | 1485000 | Official data |
| Bhutan | Asia | 531 | Official data |
| Brunei | Asia | 2319 | Official data |
| Cambodia | Asia | 651945 | FAO data based on imputation methodology |
| China | Asia | 27116250 | FAO estimate |
| East Timor | Asia | 125760 | FAO data based on imputation methodology |
| Georgia | Asia | 18361 | FAO data based on imputation methodology |
| India | Asia | 114151770 | FAO data based on imputation methodology |
| Indonesia | Asia | 894278 | Official data |
| Iran | Asia | 199000 | (17) |
| Iraq | Asia | 300000 | (18) |
| Jordan | Asia | 95 | FAO data based on imputation methodology |
| Kazakhstan | Asia | 10414 | FAO data based on imputation methodology |
| Laos | Asia | 1200040 | Official data |
| Malaysia | Asia | 117707 | FAO data based on imputation methodology |
| Myanmar | Asia | 3790031 | FAO data based on imputation methodology |
| Nepal | Asia | 5277819 | Official data |
| Pakistan | Asia | 38848000 | Official data |
| Philippines | Asia | 2882655 | Official data |
| Sri Lanka | Asia | 308790 | Official data |
| Syria | Asia | 8000 | FAO estimate |
| Tajikistan | Asia | 15351 | FAO data based on imputation methodology |
| Thailand | Asia | 1258272 | FAO data based on imputation methodology |
| Turkey | Asia | 161439 | Official data |
| Vietnam | Asia | 2425105 | Official data |
| Hong Kong | Asia | 329 | FAO data based on imputation methodology |
| Taiwan | Asia | 2057 | Official data |
| Albania | Europe | 95 | FAO data based on imputation methodology |
| Bulgaria | Europe | 12809 | Official data |
| Germany | Europe | 9613 | Official data |
| Greece | Europe | 9239 | FAO data based on imputation methodology |
| Hungary | Europe | 1000 | (19) report herd count, (20) report herd still exist in the country |
| Italy | Europe | 402796 | (21) |
| Kosovo | Europe | 400 | (22) |
| North Macedonia | Europe | 643 | FAO data based on imputation methodology |
| Poland | Europe | 69 | (23) |
| Romania | Europe | 14000 | (24) |
| Russia | Europe | 5311 | Official data |
| Serbia | Europe | 1000 | (25) |
| Switzerland | Europe | 1200 | (26) |
| United Kingdom | Europe | 2500 | (27) |
| Ukraine | Europe | 120 | (28) |
| Australia | Oceania | 133000 | Lemcke, 2019 *apud* Zhang (29) |
| Guam | Oceania | 94 | FAO data based on imputation methodology |
| Micronesia | Oceania | 173 | FAO data based on imputation methodology |
| Papua New Guinea | Oceania | 3500 | (30) |
| New Zealand | Oceania | 1000 | Estimated according to farms website available |

*The official data and the FAO data were obtained at FAO(1).

**References**

1. FAO. FAOSTAT. (2019) Available at: http://www.fao.org/faostat/en/#data/QA [Accessed January 19, 2020]

2. Wilson RT. The Domestic (Water) Buffalo in Africa: New and Unusual Records. *J Buffalo Sci* (2016) **5**:23–31.

3. Rademeyer W. Buffalo Ridge head count. (2020)

4. ABUAR. Asociación para el Desarrollo y Producción del Búfalo en Argentina. Buenos Aires (2019).

5. Zava M. Developments of Buffalo Industry in America. *Buffalo Bull* (2013) **32**:75–82.

6. ASOBUFALOS. Asociación Colombiana de Criadores de Búffalos. Medellín (2019).

7. Hernández-Mora G, Bonilla-Montoya R, Barrantes-Granados O, Esquivel-Suárez A, Montero-Caballero D, González-Barrientos R, Fallas-Monge Z, Palacios-Alfaro JD, Baldi M, Campos E, et al. Brucellosis in mammals of Costa Rica: An epidemiological survey. *PLoS One* (2017) **12**:e0182644. doi:10.1371/journal.pone.0182644

8. Obregón D, Cabezas-Cruz A, Armas Y, Silva JB, Fonseca AH, André MR, Alfonso P, Oliveira MCS, Machado RZ, Corona-González B. High co-infection rates of Babesia bovis, Babesia bigemina, and Anaplasma marginale in water buffalo in Western Cuba. *Parasitol Res* (2019) **118**:955–967. doi:10.1007/s00436-018-06194-6

9. AGLYGec. Asociación de Ganaderos del Litoral y Galápagos. Guayaquil (2019).

10. Zava MA. Personal communication. (2019)

11. ASOBUFALOS. Asociacion Gutemalteca de Criadores de Bufalos de Agua. Guatemala (2019).

12. Molina S. Personal communication. (2019)

13. AMEXBU. Asociación Mexicana de Criadores de Búfalos. Mexico city (2019).

14. APACRIBU. Asociación Paraguaya de Criadores de Búfalos. (2019).

15. American Water Buffalo Association. Water Buffalo Fact. (2019) Available at: http://americanwaterbuffaloassociation.com/Water Buffalo Fact.htm [Accessed January 18, 2020]

16. Gutiérrez-Añez JC. Current status and future challenges of buffalo production post-centenary of its introduction in Venezuela. *Buffalo Newsl* (2019)29–36.

17. Rahmaninia J, Mokhber M, Shahrbabak HM. Morphological study in azeri and khuzestani buffaloes of Iran. *Buffalo Bull* (2019) **38**:1–10. Available at: http://158.108.80.26/kuojs/index.php/BufBu/article/view/2398 [Accessed April 22, 2020]

18. Al Fartosi K. Buffalo in Iraq. in *The first International Sci. Conference for Mesopotamian Buffalo* (Thi Qar, Iraq).

19. Barna B, Holló G. The breeding of buffalo in Hungary. *Anim Welfare, Ethol Hous Syst* (2012) **8**:19–35. Available at: https://www.cabdirect.org/cabdirect/abstract/20123197653# [Accessed April 10, 2020]

20. Hornok S, Sugár L, Fernández de Mera IG, de la Fuente J, Horváth G, Kovács T, Micsutka A, Gönczi E, Flaisz B, Takács N, et al. Tick- and fly-borne bacteria in ungulates: The prevalence of Anaplasma phagocytophilum, haemoplasmas and rickettsiae in water buffalo and deer species in Central Europe, Hungary. *BMC Vet Res* (2018) **14**:98. doi:10.1186/s12917-018-1403-6

21. CRenBuf. Traceability of the buffalo supply chain system. *MIPAFF MINSA Ist Zooprofilattico Sper del Mezzog* (2019) Available at: http://90.147.124.11/index.php?page=home [Accessed May 28, 2020]

22. Bytyqi H, Mehmeti H. Buffalo populations and their breeding in Kosovo - International Buffalo Federation. (2015) Available at: https://internationalbuffalofed.org/buffalo-populations-breeding-kosovo/ [Accessed January 18, 2020]

23. Kobak P, Pilarczyk B. Prevalence of gastrointestinal parasites of water buffaloes raised in the Notecka forest region (Poland). *Bull Vet Inst Pulawy* (2012) **56**:33–36. doi:10.2478/v10213-012-0006-4

24. Popa R, Popa D, Vidu L, Pogurschi E, Maftei M, Nicolae C. Economic weight of production traits for Romanian buffalo. *Sci Pap Ser D, Anim Sci* (2018) **61**:35–40. Available at: http://animalsciencejournal.usamv.ro/pdf/2018/issue_1/Art6.pdf

25. Perisic P, Bogdanovic V, Mekic C, Ruzic-Muslic D, Stanojevic D, Popovac M, Stepic S. The importance of buffalo in milk production and buffalo population in Serbia. *Biotechnol Anim HusbandryBiotehnologija u Stoc* (2015) **31**:255–263. doi:10.2298/bah1502255p

26. Lechmann J, Bachofen C, Ackermann M. Investigation into the virome of Swiss water buffaloes. (2016). Available at: https://www.aramis.admin.ch/Default.aspx?DocumentID=24577&Load=true [Accessed January 19, 2020]

27. Borghese A. *Buffalo livestock and products*. Rome: CRA, Council Research Agriculture (2013).

28. Dzitsyuk V V., Typylo HT. Frequency of Chromosomal Aberrations in Somatic Cells of Ukrainian Buffaloes (Bubalus bubalis L.). *Cytol Genet* (2020) **54**:111–115. doi:10.3103/S009545272002005X

29. Zhang Y, Colli L, Barker JSF. Asian water buffalo: domestication, history and genetics. *Anim Genet* (2020) **51**:177–191. doi:10.1111/age.12911

30. Presicce GA. *The Buffalo (Bubalus bubalis) - Production and Research*. Sharjah: Bentham Science Publishers (2017). doi:10.2174/97816810841761170101
